# Reduplication of Both Sounds of the Heart

**Published:** 1855-06

**Authors:** Austin Flint

**Affiliations:** Professor of the Theory and Practice of Medicine in the University of Louisville


### BUFFALO MEDICAL JOURNAL

AND

### MONTHLY REVIEW.

VOL. 11. June ~~MAY~~, 1855. NO. 1.

### ORIGINAL COMMUNICATIONS.

ART. I.—*Reduplication of both sounds of the Heart. Case and Remarks.* By Austin Flint, M. D., Professor of the Theory and Practice of Medicine in the University of Louisville.

*Case.* George Nash, aged twenty-seven, an Englishman, boatman, was admitted into the Louisville Marine Hospital, Dec. 16, 1853. The following account of the case is condensed from the hospital record:

*Previous History.* The patient states that he has been a sailor for nine years. Prior to eighteen months ago he had good health. Eighteen months ago he had epidemic cholera, with which he was confined eighteen days. After this attack he recovered, apparently, his former health, and continued well up to six weeks ago, when he thinks he contracted a severe cold, being attacked with cold, accompanied by a sense of soreness beneath the sternum. The cough has continued to the present time, and been more troublesome for the past fortnight than previously. It has been especially troublesome at night. Has had no medical advice prior to his admission, and has taken no remedies except a few cough lozenges. Has never had hemoptysis, nor chills. His appetite has been good, and bowels regular.

*Present Condition.* The aspect of the patient is not morbid. Pulse normal. Respiration slightly increased in frequency. Skin and tongue normal; bowels regular; appetite good; no thirst. Percussion sound clear over chest on both sides. Breathing movements on the two sides equal. Sibilant *râle* heard with both acts of respiration on both sides.

The foregoing account was noted by Dr. S. M. Dickinson, resident physician at the hospital. On the 23d December, seven days after his admission, the following record was made by me:

He reports improvement since his admission. The cough is still troublesome, but there is less soreness in the chest, and the quantity of expectoration has diminished. The treatment has consisted of a mixture of syrups, ipecacuanhæ, scillæ and tolutani, with a little of the sulphate of morphia. He thinks he has gained strength since he entered the hospital. He sits up the entire day. He says he has never had rheumatism, and does not recollect ever having had any acute disease attended with pain, or other prominent symptoms referrible to the chest. He has noticed that for the last eight or ten years he has been a little short-winded, but, to quote his language, "nothing worth speaking of." Has considered himself a healthy man, able to perform any kind of manual labor, till within a few weeks past. He now experiences considerable embarrassment of breathing on exercise. Sleeps badly at night; suffers from a sense of fullness within the chest, want of breath, and has bad dreams. He has been conscious of palpitation of the heart since he contracted his present cold. He is obliged to lie with his head elevated at night, else he suffers from dyspnœa, and is troubled with cough. This he states to have been the case only since he took cold. The general aspect is not morbid, but there is slight lividity of the prolabia. He weighed one hundred and eighty-five pounds ten days before his admission. His pulse cannot be appreciated at the wrist sufficiently for enumeration, but the carotids can be felt, and, enumerating in this situation, counting, at the same time the heart sounds, the number of ventricular contractions, per minute, is one hundred and sixty! There is no œdema of the lower extremities, and no ascites. The least exertion, particularly with stooping, occasions panting and injection of the face.

*Physical Signs.* The chest is well developed. The pectoral muscle is very large. On percussion in precordia, transversely in line of nipple, flatness extends to the nipple, and dullness for some distance beyond it. The respiratory murmur in front is vesicular, and appears, on brief examination, to be equal on both sides, but somewhat more evolved at the summit of the left side. There is no apex impulse of the heart. A feeble diffused impulse is felt just below the nipple, and both seen and felt in the epigastrium. There is no heaving of the chest. The heart sounds succeed each other so rapidly that it is difficult to study them, but they appear to be pure, and the first sound is shorter than natural.

*Dec.* 24. Capillary congestion is considerable on the hands, and slightly over the remainder of the upper extremities, and the body generally. Pulse at the wrist too indistinct to be counted, but, *counted on the neck, it is one hundred and sixty.* He perspires freely at night.

*Physical Signs.* An impulse is seen and felt in the epigastrium, and a feeble, diffused impulse in precordia. The sounds of the heart are heard more distinctly below, than above the pectoral muscle (the muscle is largely developed.) They are also heard quite distinctly at the epigastrium. The sounds are regular, no variation in rhythm occurring during the examination. Below the pectoral muscle, and at the epigastrium, a short, sharp *bruit* is appreciable. *No pulsation of jugular veins.* The jugular veins not visible, nor the veins on the hands or elsewhere.

*Dec.* 28. On examination of this patient yesterday, I found a feeble vibratory pulse at the wrist, numbering eighty per minute. I counted it repeatedly with similar results. On counting the heart sounds, I found them apparently one hundred and sixty-six beats, *i. e.* tic-tacs, per minute. I repeated the comparison of the pulse with the heart sounds several times with the same results. To-day I find the same contrast, viz., pulse eighty, and two heart sounds repeated one hundred and sixty times per minute. Dr. Dickinson counted, without knowing the results of my enumerations, with similar results.

*Dec.* 29. Repeated the enumeration of the pulse and heart sounds, with the same results as yesterday. On directing attention to the point, every alternate systolic sound seems louder. Lest it may be supposed that, in counting at the wrist, I enumerated the pulsations in my own finger, I state that I find my own pulse directly after the foregoing enumerations, to be ninety-six.

*Dec.* 31. Dr. Hardin, of Louisville, now Professor of Midwifery in the Kentucky School of Medicine, happening to be in the ward this morning, counted the pulse of this patient, and found it seventy-six. I counted directly the heart sounds, and found the number of apparent beats, or double sounds to be one hundred and fifty-two. The ratio it will be observed, between the two is preserved, viz., two heart sounds occurring precisely twice for a single pulse at the wrist.

*Jan.* 1, 1854. The pulse at the wrist, and the heart sounds have been counted by Dr. John Clark, of Louisville, without any information given him of the case, he reported as follows: pulse at wrist, seventy-nine; two heart sounds, one hundred and fifty-eight.

*Jan.* 3. *Physical Signs.* Below pectoral muscle, with the systole, a faint, short, sharp *bruit* is appreciable. It is heard at the apex—alike at the right and left apex. At the base no *bruit* is perceptible, and the heart sounds are extremely feeble in that situation. There is a diffused impulse over the whole precordia, but no heaving. The pulse, to-day, is very feeble, scarcely appreciable, and bears the same ratio to the heart sounds as hitherto.

He continues to complain of cough, and of being put out of breath when the paroxysms of coughing are violent. He is, however, capable of pretty active muscular exercise without much inconvenience. For example, this morning he turned out, with some other inmates of the ward, to pump water into the reservoir on the top of the building. He said he felt the want of breath after pumping for some time, but not at once. He continues to present a normal aspect. There is no œdema.

*Jan.* 13. Pulse very feeble, scarcely appreciable, eighty per minute. Heart sounds one hundred and sixty. The diastolic sound scarcely appreciable on mediate auscultation. There is no heaving impulse, but a shock, or blow communicated to the ear. The first sound has a ringing, vibratory quality (*bruit de moulin*). A visible slight movement is apparent in the precordia with each systole. At the base of the heart no *bruit* accompanies the heart sound. At the right base the sounds are distinctly defined, their relative characters preserved; at the left base they are less defined. At the left apex a *bruit*, supposed to be endocardial, accompanies the systole, uniformly higher in pitch than the whispered word *who*, and nearly as high as *R.* The same sound is heard at the left apex, somewhat louder than at the right, the pitch being the same. The diastolic sound is not appreciable at the apex. The *bruit* is only appreciable at the apex, not over the body of the organ.

He still complains of paroxysms of cough during the night, attended with dyspnœa. The cough is unattended with expectoration. He is annoyed at times, especially in the morning, with palpitation. He is up and about the ward all day, and thinks, on the whole, that he is improving.

*Jan.* 25. Continues to suffer much during the night from dyspnœa and cough. For a week past puffiness under the eyes has been observed, and within the last three days the lower extremities have become œdematous. There is pitting on pressure as high as the knees. Last night he was obliged to maintain the sitting posture on account of the dyspnœa.

*Jan.* 31. He is obliged to sit up much of the night on account of dyspnœa. During the day, for the most part, he is free from this difficulty. There is moderate œdema of the feet and legs. Pulse is scarcely appreciable, and exceedingly irregular.

*Feb.* 4. The following record was made by Dr. Dickinson: On the appearance of œdema the super-tartrate of potash was prescribed, and continued for several days; and then the sulphate of magnesia in purgative doses. The quantity of urine became increased, and he expressed considerable relief after being purged. There is evidently liquid effusion within the peritoneal sac; the scrotum is œdematous, and the entire lower extremities, but no evidence of hydro-thorax. He cannot maintain long the recumbent posture, and slight exercise occasions dyspnœa.

A few days after the foregoing date I left Louisville. On the 14th of February the following record was made by Dr. Dickinson: This patient was discharged to-day at his request. He reports feeling quite well. He is now able to sleep without difficulty in the recumbent posture. He experiences no trouble from active exercise. He has taken occasional purgative doses of the sulphate of magnesia, and of the syrup of the iodide of iron thirty drops, twice daily, since the date of the last record. Has had full diet. The œdema and ascites have disappeared. His aspect is normal; appetite good; no cough nor pain in chest; no palpitation. *The pulse and the two sounds of the heart are eighty-four per minute.* Marked dullness of the percussion sound extends to the left of the nipple. No point of apex impulse is seen or felt, but a very feeble impulse is appreciable over an area about two inches in diameter. No *bruit* heard.

*April* 12. The following record was entered by Dr. Dickinson: This patient left the hospital at the date of the last record, and engaged as a deck hand on a steamboat for New Orleans. He was absent three weeks, and he states that, during that time, he worked as hard as ever before in his life, and never had better health. On his return to Louisville he entered the hospital as a ward attendant. No examination has been made since his return until this morning, when he has been examined by Dr. Hardin and myself with the following results: Pulse at the wrist eighty-four per minute and normal; systolic ventricular contractions, as determined by the heart sounds in precordia, eighty-four per minute. Sounds of heart pure. No apex impulse appreciable. Slight elevation of precordia. Dullness on percussion extends an inch to the left of the nipple. General health good.

Up to the time I am writing, February 13, 1855, I have not succeeded in seeing the subject of this case, and making an examination of the chest. I have learned, however, that he has been in the vicinity of Louisville during the past summer, autumn and winter, and apparently enjoyed excellent health, having been, and being now capable of active labor. I hope to be able to append to this report an account of his present condition as determined by existing physical signs.*

*Remarks.* The foregoing account, although considerably condensed from the hospital record, embraces details which would be tedious were the case not one of unusual interest. As it is, on reviewing the record, there is less occasion to complain of its prolixity, than to regret the absence of particulars which more minute investigation might have developed. Several points suggest themselves concerning which the notes are defective, and which, were an opportunity to observe a similar case to be again offered, would claim attention. As regards my own clinical experience, the case was *unique*. I was practically unacquainted with the subject of reduplication of the heart sounds. The explanation which, at the time, I adopted as probably correct was, that the double sounds, compared with the pulse at the wrist, involved an equal number of veritable systoles and diastoles of the heart—in other words that the veritable beats of the heart were really twice as frequent as the pulse at the wrist, the force of every alternate contraction of the left ventricle being insufficient to produce an appreciable dilatation of the radial artery. In reflecting upon the subject subsequently, without referring to the record, I was led to form a different opinion, viz., that the reduplication was due to a want of synchronism in the contraction of the two ventricles. This theory seemed the more plausible, and with this speculative view I commenced writing the report of the case. I had forgotten that the pulsation

* Feb. 26. After finishing this paper, I succeeded in seeing the subject of the case which I have reported. His aspect denotes robust health, and he declares that he was never in better physical condition than at the present time. He can take active exercise without any embarrassment of respiration ; is never troubled with palpitation ; has no cough, and in a word considers himself in all respects a healthy man. There is dullness over an abnormal area in precordia ; no impulse is appreciable ; no bellows murmur ; the sounds of the heart are pure, regular in rhythm, and their relation to the pulse normal. In short, no physical evidences of heart disease are found save that the area of dullness is somewhat increased.

of the carotid artery was equal to the number of the heart beats. This fact which appears in the record, must, probably, be considered, incompatible with that theory. The question, then arises, what is the true explanation? Before offering any answer to this question, it is proper to inquire whether important facts bearing on the rationale of reduplicated heart sounds are to be found in treatises on diseases of the heart, and, also, what opinions are entertained on the subject by those who have given special attention to the study of these diseases.

Cases of reduplication of the heart sounds are certainly not of frequent occurrence, and do not appear, as yet, to have been studied with much success. M. Bouillaud claims to have been the first to describe this peculiar aberration. In a late publication, *Leçons cliniques sur les maladies du coeur etc., par M. Bouillaud, recueillies et rédigé par C. D. V. Racle*, 1853, he says that, occasionally, in place of the normal tic-tac, and of the adventitious sounds (bellows murmurs) are found *three*, and even *four* distinct sounds produced during a single beat or revolution of the heart. He adds that he was able to cite but a single instance in the first edition of his treatise on diseases of the heart in 1834; but before the appearance of the second edition in 1841, he had collected several examples, so that the reality of the phenomena could no longer be denied, as it had been by some, and, moreover, it had been, in the meantime, abundantly confirmed by other observers.

The reduplication, as just stated, may affect but one of the heart sounds, causing three sounds with every beat; or both sounds may be doubled, giving rise to four sounds with each beat. The systolic or diastolic sound may be alone doubled, the latter occurring much oftener than the former. Then, in place of the normal *tic-tac*, the three sounds may be represented as follows: systolic reduplication, *tic tic tac;* diastolic reduplication, *tic tac-tac*. With the same mode of illustration, when both sounds are repeated the representation is, *tic-tic tac-tac.**

On referring to the second edition of the treatise by M. Bouillaud, the histories of twelve cases of different varieties of reduplication will be found. Of these twelve cases, in five the condition of the heart after death was ascertained. In six of the cases the diastolic sound was doubled; in one case the systolic sound, and in one case both sounds. In the four remaining cases but one sound was repeated, but it is not stated whether it was the systolic

* The words tic-tac are employed here as serving the present purpose as well as others which in other respects than the rhythmical succession of the sounds, are undoubtedly to be preferred. Reversing the order, viz., tac-tic, is a more faithful representation.

or diastolic. In each of the twelve cases there was physical evidence of valvular lesions involving contraction either at the mitral or aortic orifice, or insufficiency, with enlargement of the heart. In the five cases in which the results of examinations after death are given, these lesions were found to exist. In three of these five cases the contraction was at the aortic, and in two at the mitral orifice.

In several of the cases a reduplication of the pulse was also observed.

The single instance in which both sounds were doubled is reported as follows:

"CASE V. A man, aged forty-five years, died Sept. 10, 1836. A triple, and afterward quadruple *bruit de soufflet* coëxisted *with a double pulsation of the arteries.**

"*Exploration*, *August* 16. Irregularity and intermittency of the pulse, which is strong and vibratory, so as to strike with a painful degree of force against the finger, numbering eighty-eight. At the same time all the arteries pulsate distinctly twice, *coup sur coup*, the second pulsation being smaller and more feeble than the first. Each of these pulsations is accompanied by a *bruit*, the intensity of which is proportionate to the corresponding pulsation. An interval longer than ordinary succeeds the second pulsation.

"The heart beats equally twice, *coup sur coup*, and each of its two systoles is accompanied by a loud *soufflet*. Thus three sounds succeed each other in a manner to imitate the beating of the drum known under the name of *rappel*.† The interval which separates the second systolic from the diastolic sound, is a third longer than that which divides the two systolic sounds. The *bruit de soufflet* which is most prolonged, is evidently synchronous with the diastole. The two pulsations, *coup sur coup*, of the heart and arteries, are visible as well as appreciable by the touch.

"*Exploration*, *August* 21–22. The diastole of the left ventricle appears to be doubled as well as the systole, and there are heard, *coup sur coup*, four *bruits de soufflet*, the two which correspond to the diastole being louder than the two others. These four successive sounds of the heart are very distinct, readily enumerated, and their existence has been verified by several observers who follow my clinique, and who are skilled in this kind of exploration.

"*Morbid Appearances*. Aortic valves shriveled, corrugated, thickened, fibro-cartilaginous, leaving a space between them when depressed capable of

---

* I have italicized the above. Its bearing on the subject will appear presently.

† Tattoo.

receiving the little finger. Considerable hypertrophy and dilatation of the left ventricle. Mitral valve thickened, but no contraction of the orifice. No contractions of the orifices at the right side of the heart."*

So far as I know, M. Bouillaud is the only author who has reported a series of cases of reduplicated heart sounds, giving, in connection with clinical observations, the morbid conditions found after death. In saying this I should add that I have not taken pains to examine numerous treatises and periodicals with a view to find accounts of cases of this description. In fact, in addition to the works of M. Bouillaud, and an article in the *Archives de Médécine*, to which reference will presently be made, I have consulted only the treatise on auscultation by Barth and Roger, (edition of 1854); the recent work on diseases of the heart and aorta by Dr. Stokes; a treatise on diseases of the heart by Dr. Bellingham, of Dublin, lately published, and the last edition of the admirable work by Dr. Walshe. A brief space is devoted to the subject by each of these authors, and theoretical explanations are offered, to which I shall presently advert. All of them have met with illustrations of reduplication of one of the heart sounds. Such instances are probably not extremely rare, but it has never fallen to my lot to meet with one prior to the last summer, when my attention was directed to a case in the Royal Infirmary of Edinburgh by Dr. Bennett. Cases of reduplication of both sounds are, however, justly entitled to be included among the rarest of the curiosities of clinical experience. Dr. Stokes states that he has never met with an instance. Barth and Roger evidently are practically unacquainted with it. Dr. Walshe implies in his language that with him it has been a matter of observation, but he does not state whether he has met with more than one illustration; but one case of this variety is contained among the cases reported by Bouillaud; and in addition to this case, two others only, so far as my knowledge extends, have been reported. One of these cases is reported in an article contained in the *Archives Générales de Médécine*, by Dr. Charcelay, of Tours, France, No. for December, 1838. This article is entitled, *Mémoire sur plusieurs cas remarquables de défaut de synchronisme des battements et des bruits des ventricules du cœur.* The other case is quoted in the same article from a thesis by M. Pressat, published in Paris in 1837. Dr. Charcelay cites the case observed by him at the Hospital *la Charité*, Paris, in proof of the occurrence of the want of synchronism in the contraction of the two ventricles. This is the explanation proposed by him, as the title of his article implies; and he professes to have observed an

* Traité Clinique des Maladies du Cœur.—Tome 2, page 348.

absence of synchronism in the contractions of the ventricles in experiments made on inferior animals. This explanation is adopted as the most rational by the several authors mentioned above.

The case observed by Dr. Charcelay presented a feature which was not present in the case now reported by me, and the peculiarity referred to, viz: pulsation of the jugular vein, has an important bearing on the question as to the mechanism by which the reduplication is produced. Moreover, the lesions found after death differed from those detailed in the case of M. Bouillaud.

The following particulars taken from Dr. Charcelay's account, are all that are important to be quoted: "The patient was a female aged 72 years, laboring under senile imbecility. There existed a little œdema of the arms. The pulse was small and irregular. There existed dyspnœa, frequent respiration, and light cough, with some mucous sputa. The chest was sonorous on percussion, except posteriorly and inferiorly. In the latter situation a very fine mucous râle was observed. Abnormal flatness on percussion existed in the precordial region without *bruit de soufflet* or *frémissement cataire.* The beating of the heart was irregular and tumultuous. Distension, bending and pulsation of the external right jugular vein, with purring thrill, *(frémissement cataire,)* were well marked; in the left jugular the thrill did not exist, and the other phenomena were less marked than on the right side. On comparing the pulse of the right jugular with that of the left carotid, it was found that the arterial preceded perhaps a little the venous pulse. The difference, however, was but slight. On applying to the precordial region a flexible stethescope which permits, at the same time that the heart sounds are auscultated, observation of the jugular vein, the carotid being pressed by the finger, it was evident that the venous and arterial pulsations, the apex impulse of the heart, the ventricular systole and the first sound, all occurred simultaneously; then followed the second sound which was clearer than the first; and after the repose succeeding the ventricular diastole, the first sound recurred, with the arterial and venous pulsation, etc.

"The foregoing took place when the movements of the heart were regular, but when they became tumultuous, at first they were so quickly developed and confused that it was impossible to analyze them; and it was not without difficulty that at length this was accomplished. On auscultating, as just mentioned, with the flexible stethescope, *it was apparent that the jugular and carotid pulsations were not constantly synchronous; that these pulsations and the first sound were not always simultaneous; that the venous pulse uniformly accompanied the first sound, while the arterial pulse fre-*

*quently failed, so that now the right ventricle, and then the left, performed the systole and diastole independently of each other, accompanied by corresponding sounds, which were sometimes increased to four in number. In the latter case, the reduplication of the beating and the ventricular sounds was complete, taking place consecutively in the right and left ventricle. But it was not generally so; oftener the reduplication was but partial, that is to say, it had reference only to one ventricle, the right ventricle, which, as it were, interposed its contractions between the simultaneous contractions of the two ventricles. The left ventricle contracted but rarely independently of the right."*

In accordance with the action of the heart, as just described, the following were the succession of events most commonly observed: a systolic sound, jugular and carotid pulsation coinciding in point of time; then a diastolic sound; after which a systolic sound, with jugular pulsation only; then a diastolic sound, and, again, a new systolic sound with a jugular and carotid pulsation, etc., etc.

Sometimes the series was as follows; 1st. Systolic sound, jugular and carotid pulsation, these always being simultaneous; 2d. Diastolic sound; 3d. Systolic sound and jugular pulsation alone; 4th. Diastolic sound—afterward, a systolic sound with carotid pulsation; and, next, a diastolic sound, followed by a return of the systolic sound with jugular and carotid pulsations, etc.

Still again, taking always as a point of departure the synchronous contractions of the two ventricles, the following was the order: 1st. Systolic sound with jugular and carotid pulsations; 2d. Diastolic sound; 3d. Systolic sound with carotid pulsation; 4th. Diastolic sound, and then contraction of the two ventricles, etc.

"In the early part of June this patient succumbed to an attack of double pneumonia."

At the autopsy the heart was found to be moderately hypertrophied without dilatation. The tricuspid valves, insufficient, presented only two fringed tongues. Its structure was dense, opaque, fibrous but not cartilaginous. Evidently when the valve was extended the orifice remained patulous. The mitral valve was healthy, as well as the sigmoid valves of the aorta and pulmonary artery. The several cavities of the heart were greatly distended with coagulated blood, as also the large vessels. The entire venous system was largely dilated; the external jugulars were voluminous, and presented a great number of bendings, the right more especially; and at the lower part of the latter there existed a varicose pouch from an inch and a half to two inches in diameter. The visceral pericardium presented some cartilaginous patches in front and behind.

The pulsation of the jugular vein, occurring without any arterial pulse and in connection with reduplication of the two heart sounds, are the combined data in the history of this case on which is based the conclusion that the movements of the two ventricles occurred independently of each other. Assuming the entire correctness of all these data, the inference drawn by Dr. Charcelay is not only rational, but none other seems admissible. If, however, there be room for doubt as to the coexistence of a reduplication of the two sounds of the heart in concurrence with the venous and without the arterial pulse, the explanation becomes dubious; in other words, the proof of the theory rests on the conditions just stated. Now in view of the statement by the author that the phenomena which he describes were developed only when the movements of the heart became tumultuous and confused, rendering an analysis of them at first impossible, and afterward difficult; and that the four sounds were but rarely heard, the reduplication generally being but partial, *i. e.* one sound only doubled, the reader cannot but feel some sense of insecurity in accepting the data necessary to the validity of the explanation.

Assuming that the reduplication of both sounds, in connection with the discrepancy between the arterial and venous pulsations, is not established with sufficient positiveness, another explanation may be offered sufficient to account for this discrepancy. It is this: the right auricle and the veins therewith connected being kept full, and distended with blood, in consequence of regurgitation through the right auriculo-ventricular orifice, it may be easily imagined that the contraction of the auricle was sufficient to occasion a venous pulsation between the ventricular systoles. The pulsation of the jugulars synonymously with the carotids is, of course, explained by the fact of free regurgitation through the tricuspid orifice.

As an exemplification of reduplication of both sounds of the heart, the foregoing case will not bear comparison with that which I have reported. In the instance observed by me, the repetition of the two sounds was as distinct as possible, the number of tic-tacs being exactly the number of arterial pulsations at the wrist whenever the latter were appreciable, and the disordered rhythm continued for days and weeks, finally abruptly ending, the patient thereafter apparently recovering good health.

The case reported by M. Pressat, and quoted in the article by Dr. Charcelay, is as follows: " A man fifty-four years of age, strong, with a sanguine complexion, presented symptoms of some heart-affection, viz; cheeks injected, lividity, hemoptysis, abdomen enlarged by the presence of liquid, lower extremities œdematous; pulse small, very irregular, numbering one hundred and sixteen; respiration twenty-eight; irregular impulse of the heart; without special *bruit;* abnormal dullness in precordia over a considerable area; demi-orthopnœa; clear percussion sound in front, over the chest; respiratory sound pure and well evolved over the right clavicle, more feeble below the left; dry, sonorous *râle* behind, quite loud, on the two sides. In this patient, who was bled, and had frictions, with digitalis internally, there was discovered, seven days after the first examination, an absence of uniformity in the beats of the heart, which had either been overlooked at the former examination, or had become developed afterward. At the left, two sounds only were heard, one clear and the other dull, but irregular and accompanied by an impulse. These sounds might be referred to the left side of the heart. At the right, two sounds, normal in their character, were heard, which might be referred to the right side of the heart. Finally, between these two situations, within a space quite limited, corresponding to the inter-ventricular and inter-auricular septs, were heard four distinct sounds, succeeding each other rapidly without interruption, like the blows of a *batteur de plâtre.* Sometimes, in place of these sounds, three only were heard, followed by an interval taking the place of the fourth, without any cause for this variation being apparent in the condition of the patient; often, also, at the right and left, the sounds of the two sides of the heart could be heard, but they were not so distinct as in the intermediate space, where it was necessary to auscultate in order to seize readily the four sounds. Some days afterward the patient feeling relieved, left the hospital."

As regards the distinct reduplication of both sounds, this case is more satisfactory than the preceding. It is probable, although not so stated, that a discrepancy between the heart sounds and pulse existed, as in the case reported by me. This, of course, must have been the fact if the reduplication was due to want of synchronism in the movements of the two ventricles, and such is the explanation adopted by the author without any hesitation. The report of the case is defective in not containing any reference to the carotid pulsation, or to the presence or absence of a jugular pulse.

In the improvement of the patient sufficiently to leave the hospital, the case bears an analogy to that which has fallen under my observation.

As already stated, the theory which explains reduplication of the heart sounds (having reference especially to reduplication of the two sounds) by supposing that the ventricles fail to contract and dilate in unison, is adopted by the several authors of recent well known works on diseases of the heart, whose nnmes have been mentioned. Dr. Walshe says: "The essential cause of these various reduplications seems to be a want of synchronism between the action of the two sides of the heart." *

Messrs. Barth and Roger say: "The formation of four sounds with a single beat has for its cause a failure of synchronism in the action of the two sides of the heart."†

Dr. Stokes transferred to his treatise the quotation just given from the work of Dr. Walshe.

Dr. Bellingham remarks: "From the manner in which the muscular fibres are arranged, it is difficult to understand how a want of synchronism could occur in a healthy heart; but if the right ventricle is much hypertrophied, and dilated, while the left remains of a normal size, such an occurrence is possible."

M. Bouillaud, in his *Leçons Cliniques*, published in 1853, regards this explanation as more probable than the one offered by himself in his treatise on diseases of the heart. To the latter explanation I shall presently refer.

The theory, abstractly considered, seems altogether reasonable, and the case reported by Dr. Charcelay, n some respects unsatisfactory, and as the reporter implies, obscured by a preconviction of the correctness of the explanation, is to be regarded as affording evidence of its correctness in proportion as the important facts, bearing on the question, which the report contains are received with confidence. The explanation would be applicable to the case which I have reported, were it not for a single circumstance which it is difficult, if not impossible, to reconcile with it, viz: the carotid pulsations sustained a normal relation to the reduplicated heart sounds, notwithstanding the discrepancy between these sounds and the pulse at the wrist. The number of carotid pulsations showed that one-half the number of sounds of the heart were due to the systole of the left ventricle, unless the pulsations of the carotid were reduplicated independently of the heart. As regards the number of pulsations of the carotid, they were ascertained and noted twice in comparison with the heart sounds. I can not believe there could have been an error of observation on this point, but I regret, in view of its importance, which I did not sufficiently appreciate at the time, that the pulse in that situation was not enumerated and noted in a larger number of instances.‡

* Diseases of the Lungs and Heart—English edition, p. 231.

† Traité Pratique d'Auscultation, etc.—Quatrième édition, 1854.

‡ In the case reported by M. Bouillaud, the arterial pulsations were doubled. His language is: " En même temps, toutes les artères battent distinctement deux fois

If not explicable by a want of synchronism in the action of the ventricles, there seems no alternative but the hypothesis first proposed by M. Bouillaud. According to this hypothesis, the systole and diastole of the ventricles take place synchronously, but are made up each of two distinct efforts (reprises.) That is to say, instead of contracting and dilating steadily, each of these two movements (contraction and dilation) are divided into two acts, and each act attended by a sound. The disorder of rhythm in this view, is analogous to interrupted or jerking respiration, called by the French writers *saccadée.* If this explanation be correct, each complete systole and each diastole were accompanied by a double sound; but the force of one only of the divided portions of the systolic contraction of the left ventricle was sufficient to produce a pulsation at the wrist, while the force of each demi-systole sufficed to produce pulsation of the carotid.

It is indeed wonderful that, of the halved portions of the systolic contraction of the left ventricle, only one should regularly be accompanied by a radial pulse; but it is perhaps quite as difficult to conceive that, in view of the arrangement of the muscular fibres of the heart, the ventricles should contract separately.

The great feebleness of the radial pulse, when appreciable, in the case which I have reported, and its absence a portion of the time, are symptoms certainly in accordance with the diminution of the power of the systole subdivided, as it is supposed to be by this hypothesis.

I do not deem it necessary to consider another hypothesis in explanation of the phenomena which I have reported, viz: that the increased number of sounds was due to nothing more than increased frequency of the heart's action; in other words, that the beats of the heart were simply multiplied, without any reduplication of the sounds. I confess this was the view which I entertained at first when the case came under my observation; and, entertaining this view, I was astonished to see a patient moving about, reporting himself tolerably well, and able to take, at times, pretty active exercise, with a heart beating one hundred and sixty times per minute! That the pulse represented exactly one half the number of systoles was sufficiently curious,

---

coup sur coup, la seconde pulsation étant toutefois plus petite et plus faible que la première." * * * "Les deux pulsations coup sur coup du cœur et des artères, sont sensibles à la vue comme à la main." When, therefore, this author in the late publication of his *Lecons Cliniques,* says that the theory of a want of synchronism is the most probable, he must either have overlooked the force of this feature of the case, or he must think there is some mode of reconciling the occurrence of two arterial pulses with a single systole of the left ventricle.

yet every practitioner knows that in cases of organic disease of the heart, and even in some instances of functional disorder, the pulse fails to represent faithfully the number of systoles. The failure occurring regularly with each alternate systole was the circumstance exciting wonder. The persistency of the multiplication of the heart sounds in a ratio compared with the pulse exactly double, for several weeks, without being associated with other symptoms denoting immediate danger, and the abrupt cessation of this irregularity, with sufficient improvement for the patient to leave the hospital, and to continue without difficulty a laborious occupation, are circumstances abundantly disproving the hypothesis last mentioned.

Finally, in view of the facts contained in the four cases of reduplication of the two sounds of the heart, which are presented in this article, it is difficult to settle upon a positive conclusion as to the mechanism by which this remarkable and rare form of disordered action is produced. The theory of a want of synchronism, so far as concerns its intrinsic plausibility, is to be preferred; but it is met, as has been seen, by a difficulty in its application to the instance occurring under my observation, and equally to the case reported by M. Bouillaud, which, unless disposed of in some way that I am not prepared to indicate, must invalidate its correctness, not only in this, but in other instances, for it is not probable that in different cases, this feature, identical in each, and so peculiar, is to be explained differently. Nor is the second hypothesis devoid of difficulties in addition to what has been alluded to, which will readily occur to the mind of the reader.

These two explanations are all that have been suggested to account for reduplication of both heart sounds, and I have no additional hypothesis to offer. An analysis of a larger number of cases may develop results which will shed more light on the subject. And in dismissing the question as to the rationale, the following quotation from the treatise on diseases of the heart by M. Bouillaud, is perhaps not less pertinent than when penned by that distinguished author more than fourteen years ago: "Je souhaite que ces faits soient fécondés par quelques uns de mes lecteurs, qui, plus heureux ou plus habiles que moi, résoudront peut-être les difficultés qu'ils me semblent encore offrir, et dissiperont les obscurités dont ils sont environnée."—*Tome* II, *page* 345.

My remarks being already more extended than I had designed, I shall not prolong this article by a discussion of the causes which occasion reduplication of one only of the sounds of the heart. I will simply say that, whatever may be the true explanation in cases in which a beat of the heart is accompanied by four sounds, the same explanation will probably apply to the instances in which the beat is accompanied by three sounds. In much the greater number of the cases of the latter description which have been reported, the second, or diastolic sound is the one doubled;* and, therefore, we may conclude, either that the two ventricles dilate separately, contracting in unison, or, that the dilation takes place, not continuously, although synchronously, but by two divided acts, each act taking place in such a manner (it must be confessed not easily understood) as to produce by a complete diastole a double sound.

Recurring to the reduplication of the two sounds, the question arises, what is its value as a diagnostic and prognostic symptom? In two of the four cases which have been given in this article, the morbid condition of the heart was determined by the post-mortem examination. The prominent lesion in each instance was different; in one consisting of aortic, and in the other of tricuspid insufficiency. In all of the four cases there was enlargement of the heart. In two cases this aberration of the heart's rhythm ceased, and improvement took place so that the patients were not obliged to remain under medical treatment; and in the case reported by me, the patient has apparently recovered good health, some enlargement of the heart doubtless remaining. In this case a *bruit* existed, supposed, but with some doubt, to be endocardial, and to denote mitral regurgitation, but it disappeared after recovery. In view of these facts it does not appear that the reduplication of the two sounds possess special significance either in diagnosis or prognosis, and it must be confessed that, with our present knowledge, the interest belonging to the subject, if the antithesis be allowable, is rather scientific than practical.

M. Bouillaud, basing his opinion on the case that had fallen under his observation, thinks that the sounds are never doubled except in connection witn organic disease involving either constriction or insufficiency. In fact, he regards the symptom as diagnostic of valvular lesions, and, inasmuch as their existence may be determined by other criteria than this, he says it is quite superfluous so far as diagnosis is concerned. He calls it "*un signe de luxe.*" The facts, however, contained in the history of my case tend to throw doubt on the correctness of this opinion. The existence of valvular disease in this case is not certain. A *bruit* supposed to be endocardial existed, it is true, but it was a matter of question, at the time, whether it was not a friction sound; and in view of its disappearance, and the present state of the

* Dr. Bellingham states the reverse of this; but he is undoubtedly in error on this point.

heart, the supposition that it was so is perhaps more probable. That a positive opinion was not formed as to the seat of the *bruit* should not subject the observer to reproach, since the most experienced auscultators have acknowledged the difficulty and indeed impossibility, in certain instances, of determining whether an adventitious sound emanating from the heart be a bellows murmur or a friction sound.

The fact that reduplication of the heart sounds is a phenomenon so rare in its occurrence, goes to show that its connection with organic lesions is only incidental; and if it be true that it may occur irrespective of such lesions, it is to be regarded as a functional disorder, which, although, perhaps, in the great majority of instances associated with structural disease, is a superadded affection. But with reference to this, as to other points, farther observations are desirable before positive conclusions are admissible.

